# Hotspots and scientometrics in gallbladder cancer surgery research: a bibliometric and visualization analysis (2014-2024)

**DOI:** 10.3389/fonc.2025.1522992

**Published:** 2025-04-03

**Authors:** Long-Fei Chen, Jian-Feng Bin, Qin Zhang, Han Li, Wei Chen, Hua Ge

**Affiliations:** ^1^ Department of Hepatobiliary Surgery, The Third Affiliated Hospital of Zunyi Medical University (The First People's Hospital of Zunyi), Zunyi, China; ^2^ Department of Gastrointestinal Surgery, The Third Affiliated Hospital of Zunyi Medical University (The First People's Hospital of Zunyi)y, Zunyi, China; ^3^ Department of Radiology, Affiliated Hospital of Zunyi Medical University, Medical Imaging Center of Guizhou Province, Zunyi, China

**Keywords:** gallbladder cancer, surgery, bibliometric analysis, laparoscopic surgery, postoperative management

## Abstract

**Background:**

Gallbladder cancer (GBC) is the most common malignancy of the biliary tract, with significant geographical variations in incidence. The prognosis of GBC is generally poor due to its aggressive nature and late diagnosis. Surgical resection is the only curative treatment, but less than 10% of patients are eligible for radical surgery.

**Methods:**

This study utilized bibliometric analysis and visualization tools to analyze research trends and hotspots in GBC surgery from 2014 to 2024. Data were collected from the Web of Science Core Collection using specific search terms related to GBC and surgical methods. The analysis was performed using tools such as CiteSpace, VOSviewer, and Microsoft Excel to identify key authors, institutions, countries, and research themes.

**Results:**

A total of 479 publications were analyzed, showing a significant increase in research output and citation frequency over the past decade. China and the United States were the leading contributors to GBC surgery research. The analysis revealed six main research clusters, focusing on early diagnosis, surgical techniques, postoperative management, and the application of advanced technologies such as laparoscopic and robotic surgery.

**Conclusions:**

The study highlights the evolution of research priorities in GBC surgery, with a shift towards minimally invasive techniques and comprehensive postoperative management. Future research should emphasize international collaboration and the exploration of emerging technologies to improve patient outcomes.

## Introduction

Gallbladder cancer (GBC) is the most common malignancy of the biliary system, accounting for 80% to 95% of biliary tract malignancies, and ranks sixth in mortality among gastrointestinal tumors (1). The incidence of GBC shows regional variation: the incidence rate in East Asia (1.4 per 100,000) is significantly higher than in developed countries like Europe (0.66 per 100,000) and North America (0.67 per 100,000) (2), which may be attributed to environmental exposure, genetic susceptibility, and inherent regional risk factors (3). Additionally, due to the unique anatomical structure of the gallbladder, the lack of distinct and specific early symptoms, aggressive growth patterns, and early lymph node metastasis, most patients are already in advanced or locally advanced stages at the time of diagnosis. This leads to a high degree of oncological malignancy and poor patient prognosis, with an average overall survival (OS) of 6 months and a 5-year survival rate of only 5% for advanced GBC (4, 5). Surgical resection of the tumor is the only treatment option that improves survival rates (1, 6), but only 10% of patients are eligible for curative resection (7). Some studies have shown that laparoscopic surgery provides patients with similar survival benefits to open surgery and better short-term outcomes (8–11), but guidelines still do not recommend laparoscopic surgery. There remains controversy regarding the extent of surgery, lymph node dissection, and other aspects for different stages of GBC (12–15).

The bibliometric method was introduced by Alan Pritchard in 1969. It allows for the rational analysis of the impact or value of research outputs by focusing on bibliographic systems and bibliometric characteristics, enabling both quantitative and qualitative analysis of the literature. The analysis process can yield detailed information on authors, keywords, journals, countries, institutions, and references within a particular research field (16, 17). This method is used to explore the productivity of researchers, institutions, and countries in specific disciplines, study research trends and focuses across different fields, and inform policy decisions (18, 19). Common bibliometric and visualization tools include CiteSpace, VoSviewer, Pajek, and HistCite. These tools and methods enhance readers’ intuitive understanding of research hotspots and frontiers in a given field. While these research methods are widely applied across various fields, there is currently a lack of bibliometric studies specific to the field of gallbladder cancer (GBC) surgery. To fill this gap and aid in resolving some of the controversies surrounding GBC surgery, this paper discusses the current state, hotspots, and frontiers of research in the field of GBC surgery, while also projecting future research trends and developments.

## Methods

### Data collection

Web of Science is a leading research platform that encompasses hard sciences, social sciences, arts, and humanities information, and is the world’s most comprehensive independent citation database (8). We enhanced the representativeness and accessibility of the data by searching the Web of Science Core Collection database, using the search terms TS =( Gallbladder cancer OR Gall bladder tumor OR The gallbladder malignancy OR GBC OR Unexpected gallbladder cancer) and TS =( Laparoscopic surgery OR Simple cholecystectomy OR Laparoscopic OR Minimally invasive surgery OR Open operation OR Laparoscopic radical cholecystectomy OR Robotic surgery). The final search was conducted on June 20, 2024, to avoid bias due to daily data updates. The search was conducted using the aforementioned terms and covered the literature published from January 1, 2024, to May 30, 2024. Excluded document types were letters, editorial materials, corrections, books, datasets, conference papers, retractions, and retracted publications. All publications meeting the inclusion and exclusion criteria, along with their complete records and cited references, were exported in plain text format.

### Bibliometric analysis

**Figure 1:**
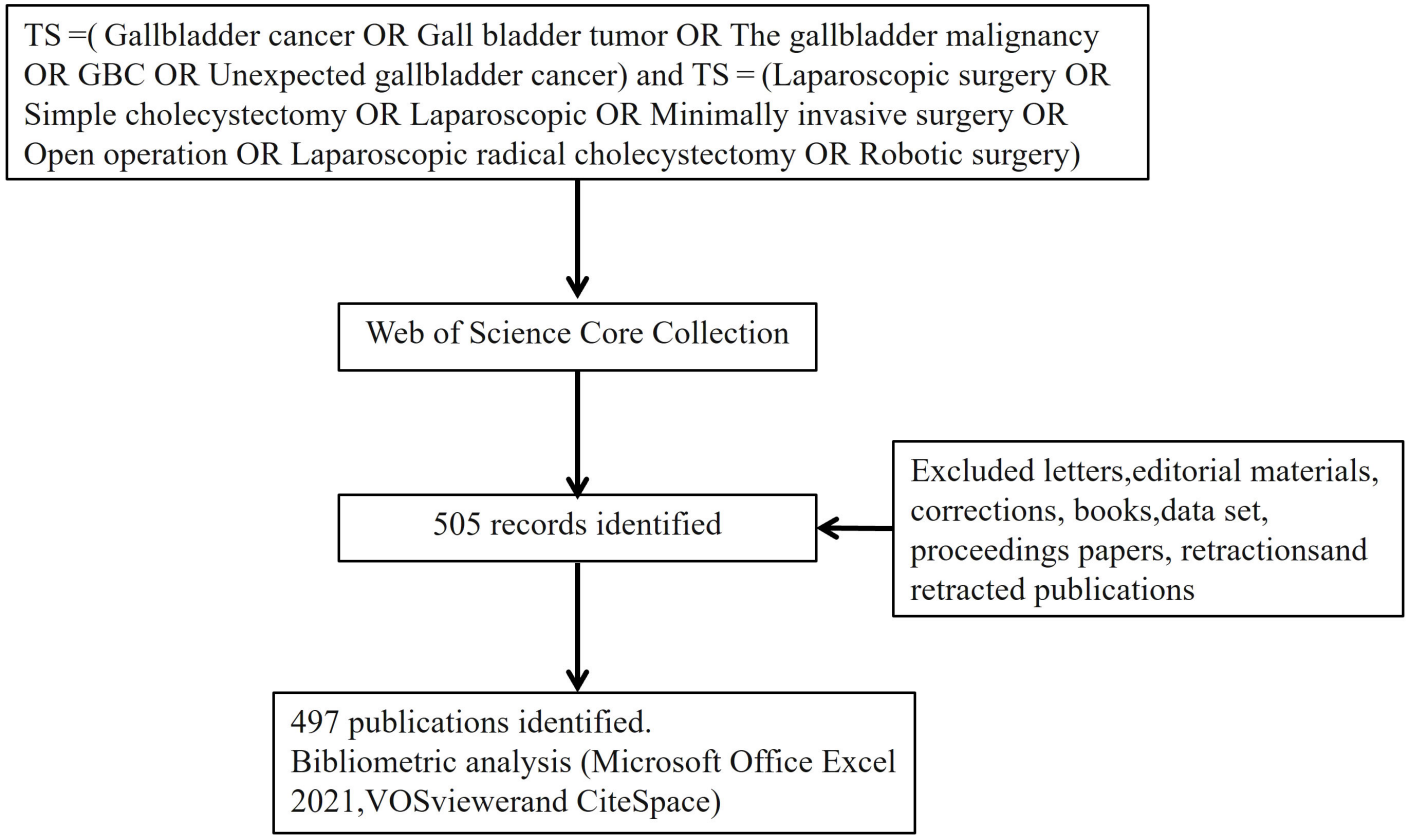
Gallbladder surgery related research into the process flow chart and ruled out.

The study conducted basic analysis using the website https://bibliometric.com, and all documents were analyzed through Microsoft Office Excel 2021, VOSviewer (v.1.6.20), CiteSpace (v.6.3), and Pajek (5.1.8). Microsoft Office Excel 2021 was used for bibliometric and error analysis of the number of publications and citation frequencies by year, and a linear prediction model based on the formula y=ax+b was established to forecast future trends in publication numbers and citation frequencies. VOSviewer is a Java-based bibliometric software developed in 2009 by the Centre for Science and Technology Studies at Leiden University, Netherlands. It has robust graphical capabilities suitable for handling large-scale data, and with VOSviewer and Pajek, keywords, institutions, journals, and countries can be visualized and clustered, generating bibliometric networks (20). We used CiteSpace to visualize keyword clusters, timelines, and their emergent burst patterns. CiteSpace, developed by Professor Chaomei Chen at Drexel University, USA, is an evolving software for document visualization analysis, used for bibliometric analysis and data visualization (21). In the study, keywords, published papers, countries, institutions, and citation frequencies were selected as nodes to construct knowledge network maps, and co-occurrence maps were used to study annual research hotspots, revealing the developmental relationships between these hotspots. **Figure 1** shows the flowchart of the search strategy and selection process used in this study.

## Results

### Temporal distribution of publications

**Figure 2:**
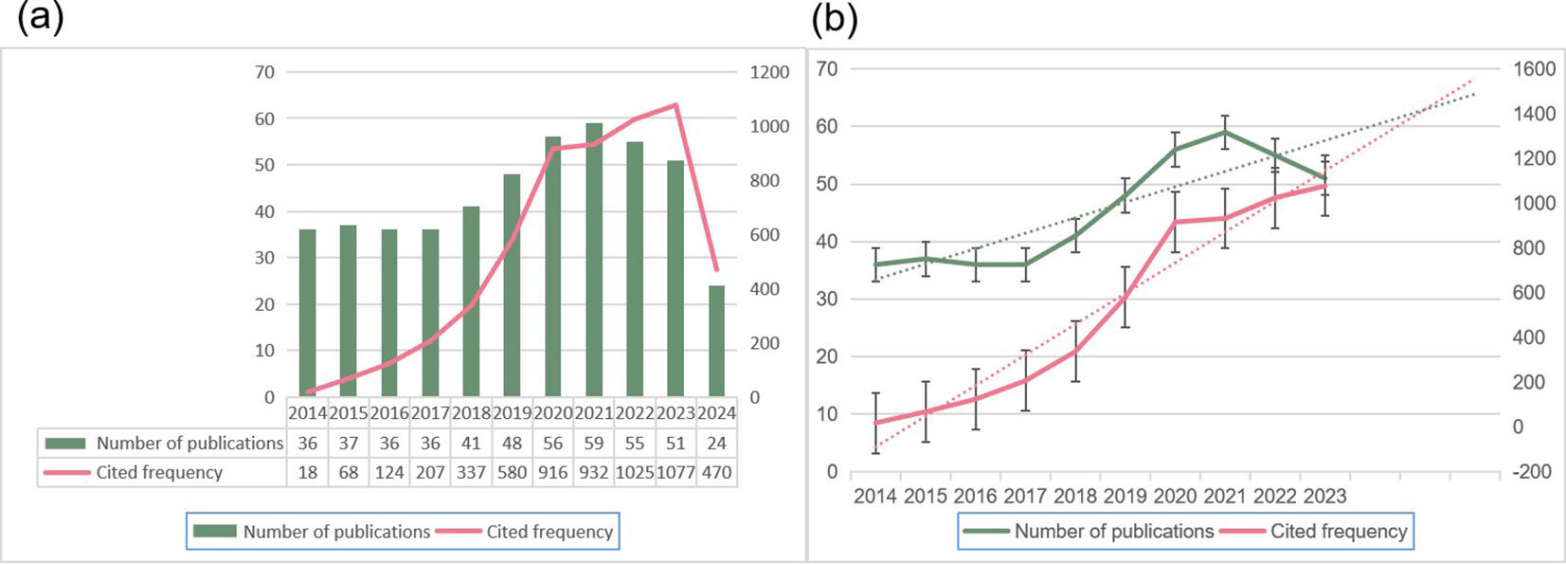
Trends in the growth of publications and citation frequency. **(A)** The number of publications and citation frequency for each year from 2014 to 2024. **(B)** Linear prediction curves for the growth trends in publication numbers and citation frequency from 2014 to 2023.

According to our search strategy, over the past 10 years, there were 505 publications related to surgery in the field of GBC, of which 479 publications were ultimately included in the study. Among these, 394 were original research articles and 85 were review articles. Since 2014, publications in this field have been recorded, indicating that it has garnered interest. However, we also found some related studies from before 2014 in other databases. Additionally, in the field of malignant tumor surgery, long-term outcomes such as 5-year survival rates or 5-year recurrence rates are commonly studied, so many projects were initiated several years ago. This also suggests that issues and controversies related to GBC surgery were already being addressed at an earlier time. **Figure 2A** illustrates the trends in the number of publications and citation frequencies in this research field from 2014 to 2024. The annual growth rate in the number of publications shows a significant increase starting from 2018, with 59 publications in 2021. Citation frequencies have increased annually since 2014, indicating a significant enhancement in the academic impact of research related to gallbladder cancer surgery. The dashed line fitting in **Figure 2B** shows that despite fluctuations, the linear prediction model still indicates an overall upward trend in the number of publications and citation frequencies, demonstrating the continued growth in research output and impact within this field.

### Contributions and distribution across different countries/regions

**Figure 3:**
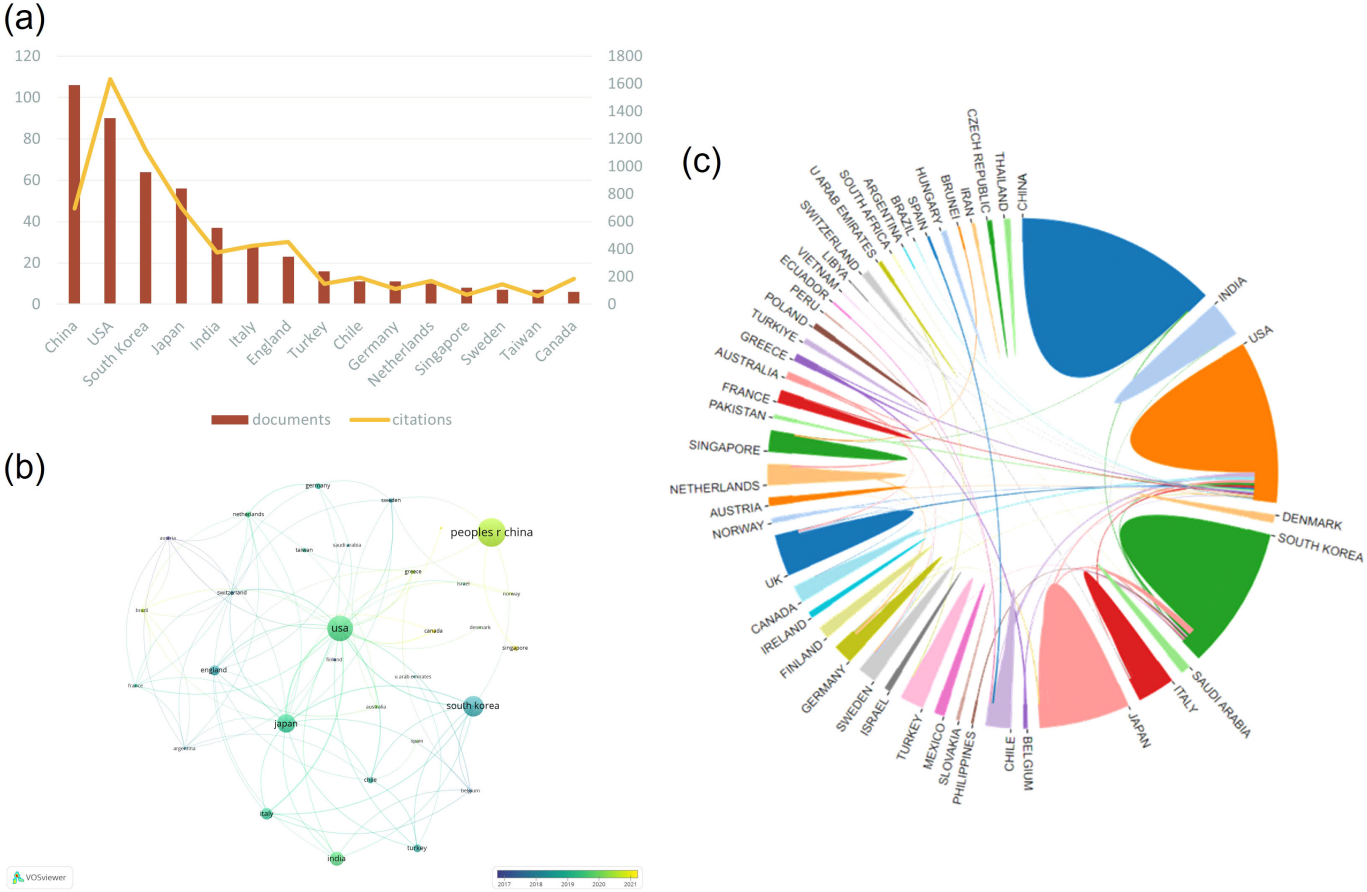
Contributions of different countries or regions to research on gallbladder cancer surgery from 2014 to 2024. **(A)** The number of publications and citation counts for the top 15 countries. **(B)** A VOSviewer network visualization map of inter-country collaborations. **(C)** Collaboration networks between countries or regions.

These publications come from 56 countries or regions, and (**Figure 3A**) shows the academic output (measured by the number of documents) and academic influence (measured by the number of citations) in this research field in multiple countries.​ This reflects that China, the United States, and South Korea dominate the majority of academic contributions and influence in this research field.

(**Figure 3B**) presents the visualization results of the global academic collaboration network. Nodes represent 56 countries each, and the size of nodes is proportional to their academic output and influence. The lines represent cooperation between countries, and the thickness and color of the lines reflect the strength and duration of cooperation. As the core node in the network, the United States maintains close cooperation with many countries, reflecting its leading position in global academic cooperation. China has a large node, and mainly has a small amount of cooperation with South Korea, Singapore, Canada and other countries. Its large number of publications shows its strong academic influence, but it is still relatively less in international cooperation and exchange. Overall, **Figure 3C** reveals the complexity and diversity of the international academic cooperation network, in which a few countries play a central role and promote global academic exchange and development through extensive cooperation.

### Institutional distribution

These publications were from 731 institutions, and the VOSviewer parameters were set as follows: methods (Linlog/modular) and minimum number of publications by institutions: 5. Twenty-four of these tissues reached the threshold. For each of the 24 tissues, the total strength of connections to other tissues will be calculated, and the one with the greatest total link strength will be selected. A list of the top 10 publications in the organizations with the highest link strength was screened (**Table 1**), listing the number of publications, the number of citations, and their total link strength for each organization.

**Table 1:** Top 10 organizations by publication volume with the highest total link strength.

Organization	Documents	Citations	Total link strength
seoul natl univ	24	567	48
yonsei univ	14	310	30
sungkyunkwan univ	13	326	42
chung ang univ	11	215	29
univ ulsan	9	267	9
Zhe jiang univ	9	38	3
Si chuan univ	8	82	0
natl canc ctr	7	204	29
niigata univ	7	166	19
gyeongsang natl univ	6	143	28

**Figure 4:**
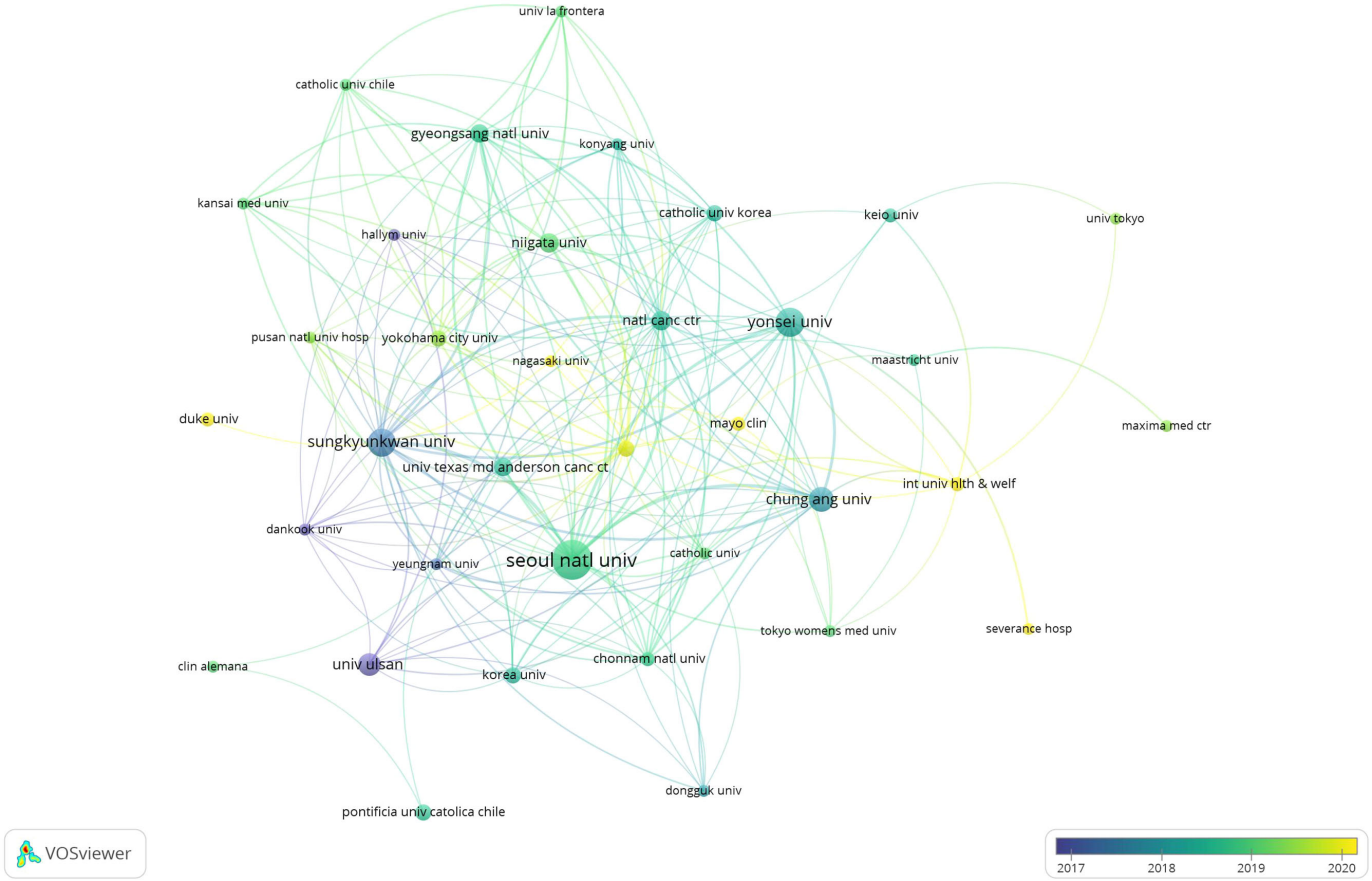
VOSviewer visual network map of different institutions’ contributions to research on gallbladder cancer surgery.

Seoul National University ranked first with 24 articles, 567 citations and 48 total link strength, followed by Yonsei University with 14 articles, 310 citations and 30 total link strength. Sungkyunkwan University ranked third with 13 articles, 326 citations and 42 total link strength. Meanwhile, the three institutions also had a strong frequency of cooperation (**Figure 4**). Zhejiang University and Sichuan University, as representatives of China, had 9 and 8 articles respectively, indicating their active degree in international cooperation. It is worth noting that China, the United States and South Korea, as the three countries with the largest number of publications, have more institutions and their affiliates in China and the United States, whose publications are relatively scattered and have not reached the screening threshold. Moreover, these institutions have relatively low link strength and low frequency of cooperation projects, so these institutions are not shown in the ranking.

### Visual network of keywords

**Figure 5:**
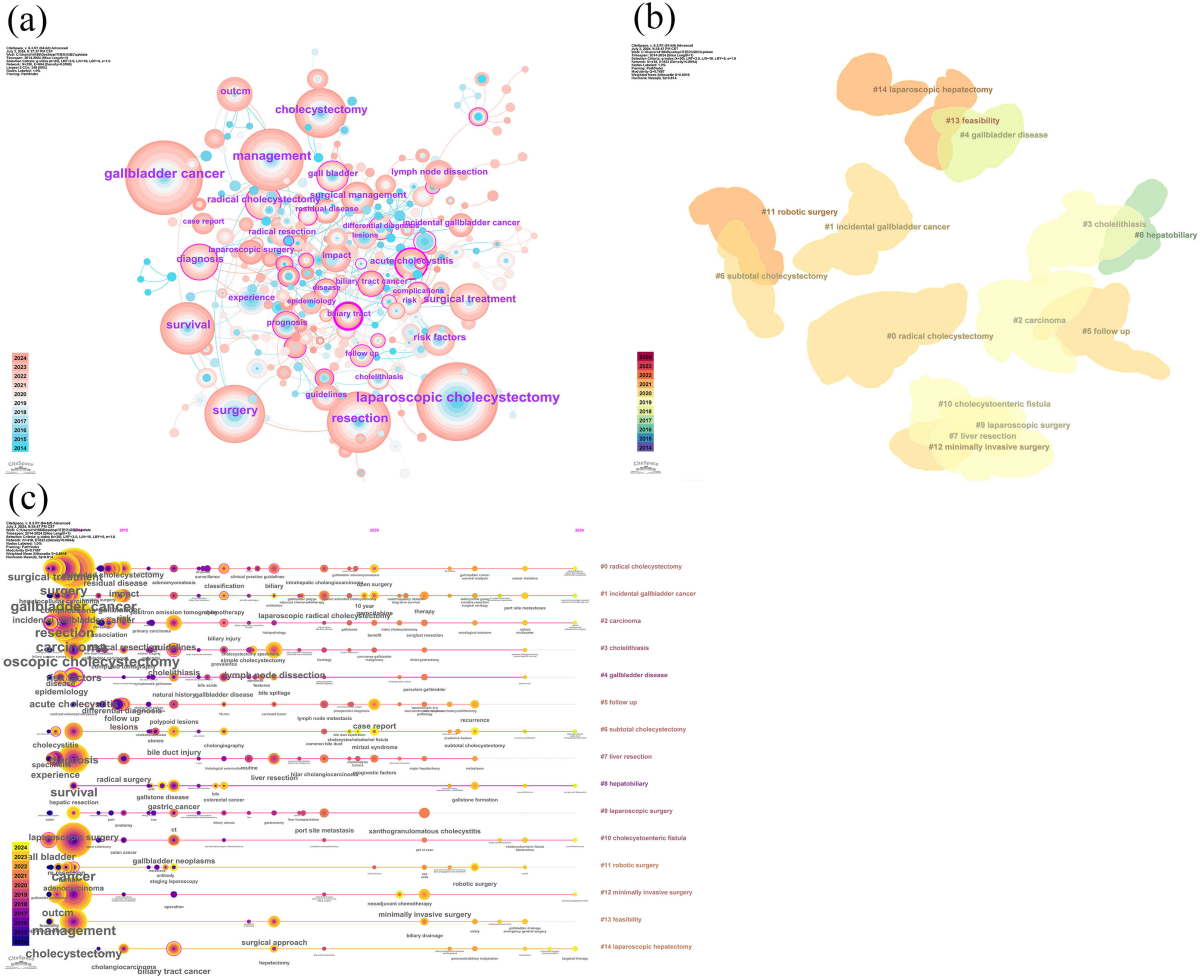
Visual network of keywords in gallbladder cancer surgery research from 2014 to 2024. **(A)** Keyword clusters in gallbladder cancer surgery research. **(B)** Keyword visualization map of the CiteSpace network in gallbladder cancer surgery research. **(C)** Timeline viewer of the VOSviewer network in gallbladder cancer surgery research.

Using CiteSpace, we constructed a co-occurrence network visualization of keywords, merging synonymous keywords, and set the g-index to k = 30 to enhance the clarity of keyword distribution. **Figure 5** illustrates the visual network of keywords in gallbladder cancer surgery research from 2014 to 2024. **Figure 5A** displays the keyword co-occurrence network in the field of gallbladder cancer surgery, with colors ranging from blue (2014) to red (2024) to indicate the temporal evolution. High-frequency keywords such as “gallbladder cancer,” “cholecystectomy,” “laparoscopic cholecystectomy,” “surgery,” and “management” form the core themes of the research. The dense connections between nodes indicate the close associations between these themes. The color changes of the keywords reflect the shift in research focus from earlier topics such as “laparoscopic cholecystectomy” and “experience” to more recent hotspots like “management,” “outcome,” and “radical cholecystectomy.” **Figures 5B, C** show the results of keyword clustering analysis and the timeline viewer in CiteSpace. The main clusters include “#0 radical cholecystectomy,” “#1 incidental gallbladder cancer,” and “#2 carcinoma,” where cluster IDs (#0, #1, #2, etc.) represent the clusters generated through the analysis. A smaller cluster ID indicates a more significant or earlier research theme, while a larger ID may indicate a secondary or emerging research direction. Larger clusters represent a greater number of members, highlighting the focus on gallbladder cancer and its surgical approaches. Minimally invasive surgical techniques (such as “#9 laparoscopic surgery,” “#11 robotic surgery,” “#12 minimally invasive surgery”) have emerged as research hotspots in recent years. The gradient color shift indicates that research hotspots have gradually moved from early diagnosis and treatment of gallbladder cancer to minimally invasive surgery and gallbladder disease management. The keyword timeline viewer demonstrates the dynamic changes in research hotspots, revealing the temporal characteristics of the research areas and the evolution of key topics reflected in the clusters.

**Figure 6:**
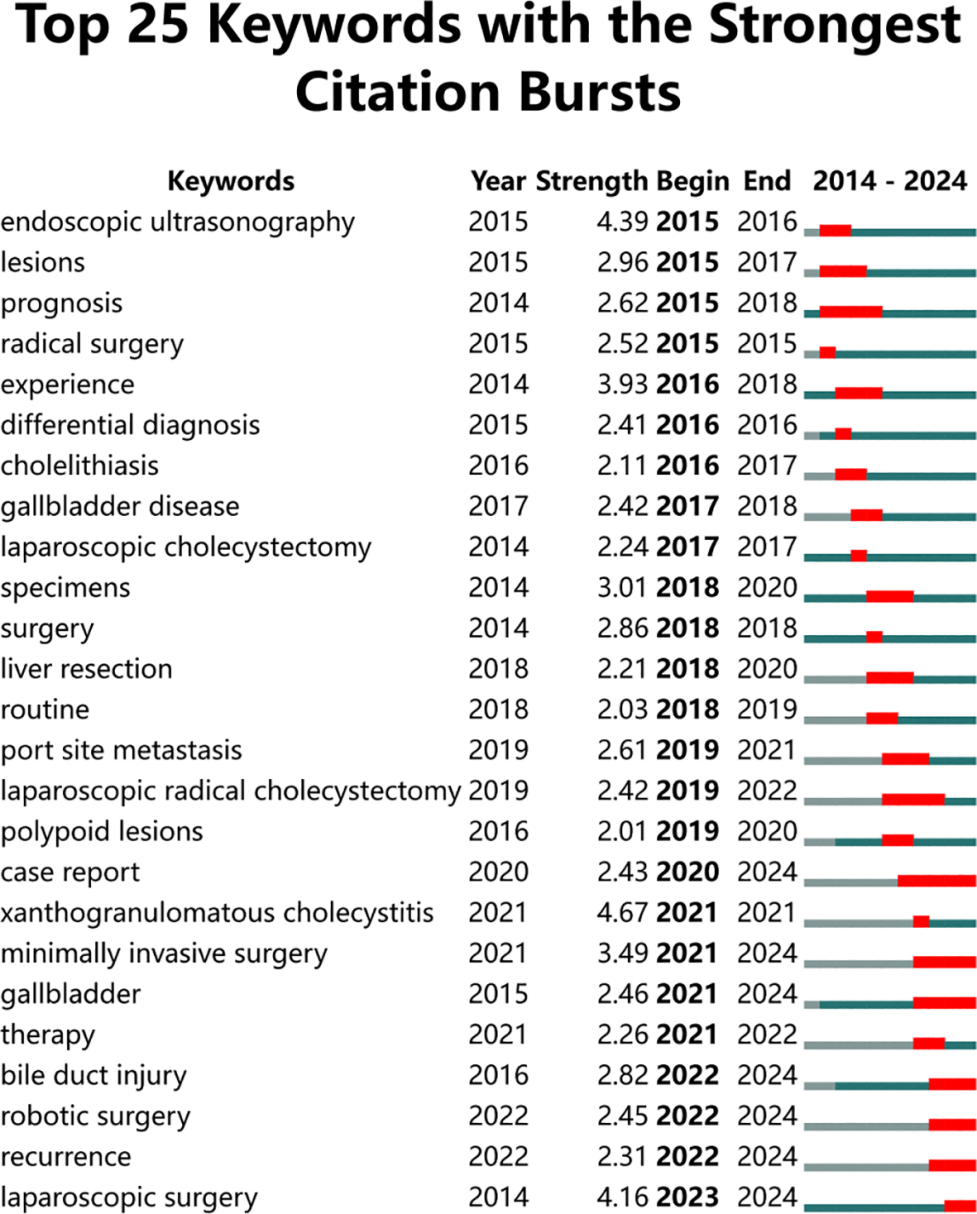
Top 25 keywords with the strongest outbreak of research related to gallbladder cancer surgery.

**Figure 6** illustrates the top 25 keywords with the highest citation bursts from 2014 to 2024, along with their citation strength and duration. The blue line represents the timeline, while the red segments on the blue timeline indicate burst detection, showing the start year, end year, and duration of the burst. The keyword “xanthogranulomatous cholecystitis” displayed the highest citation burst strength (4.67) in 2021, followed by “endoscopic ultrasonography” (4.39) and “laparoscopic surgery” (4.16). The research topics cover surgical techniques (e.g., “laparoscopic surgery,” “robotic surgery”), diagnostic methods (e.g., “endoscopic ultrasonography,” “differential diagnosis”), disease types (e.g., “xanthogranulomatous cholecystitis,” “gallbladder disease”), and clinical management (e.g., “prognosis,” “therapy,” “recurrence”). The citation bursts of these keywords reflect the dynamic evolution of research hotspots, gradually shifting focus from early concerns on diagnosis and prognosis to minimally invasive techniques and comprehensive disease management.

### Distribution of journals and the top 10 most cited publications

**Figure 7:**
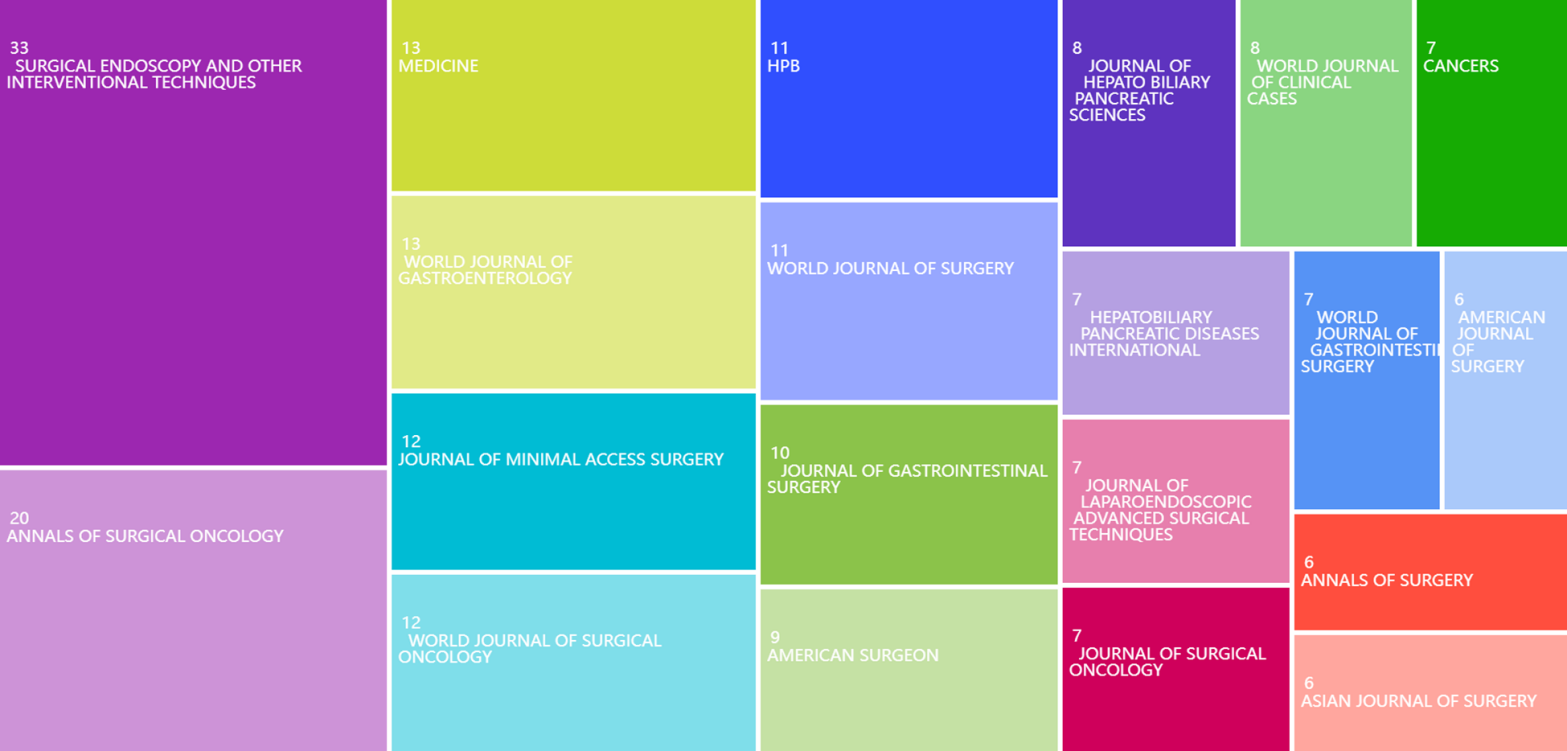
Treemap of the top 20 journals by publication volume in gallbladder cancer surgery research from 2014 to 2024.

**Figure 7** shows the number of papers published in various journals in the field of gallbladder cancer surgery and their relative impact. Surgical Endoscopy and Other Interventional Techniques (IF: 2.4) ranks first with 33 papers, indicating its significant role in this field. Annals of Surgical Oncology (IF: 3.4) published 20 papers, also showing considerable influence. (Impact factors are from the 2024 JCR)

**Table 2** presents the top 10 most cited papers related to gallbladder cancer surgery from 2014 to 2024. The most cited paper is by Choi JH et al., published in 2014 in Endoscopy, titled “Long-term outcomes after endoscopic ultrasonography-guided gallbladder drainage for acute cholecystitis,” with a total of 117 citations. The second most cited paper is by Gunasekaran G et al., published in 2020 in Hepatology, titled “Surgical Treatments of Hepatobiliary Cancers,” with 103 citations. Another highly cited paper is by Madani A et al., published in 2022 in Annals of Surgery, titled “Artificial Intelligence for Intraoperative Guidance Using Semantic Segmentation to Identify Surgical Anatomy During Laparoscopic Cholecystectomy,” with 97 citations.

**Table 2:** Top 10 most cited publications in gallbladder cancer surgery research from 2014 to 2024.

Title	Authors	Journal	Publication year	Total citations
Long-term outcomes after endoscopic ultrasonography-guided gallbladder drainage for acute cholecystitis	Choi, JH	ENDOSCOPY	2014	117
Surgical Treatments of Hepatobiliary Cancers	Gunasekaran, G	HEPATOLOGY	2020	103
Artificial Intelligence for Intraoperative Guidance Using Semantic Segmentation to Identify Surgical Anatomy During Laparoscopic Cholecystectomy	Madani, A	ANNALS OF SURGERY	2022	97
Gallbladder Cancer Diagnosis, Surgical Management, and Adjuvant Therapies	Hickman, L	SURGICAL CLINICS OF NORTH AMERICAarrow_drop_down	2019	95
Beyond the learning curve: incidence of bile duct injuries following laparoscopic cholecystectomy normalize to open in the modern era	Halbert, C	SURGICAL ENDOSCOPY AND OTHER INTERVENTIONAL TECHNIQUESarrow_drop_down	2016	92
Minimally invasive versus the conventional open surgical approach of a radical cholecystectomy for gallbladder cancer: a retrospective comparative study	Agarwal, AK	HPB	2015	85
Is Laparoscopy Contraindicated for Gallbladder Cancer? A 10-Year Prospective Cohort Study	Yoon, YS	JOURNAL OF THE AMERICAN COLLEGE OF SURGEONSarrow_drop_down	2015	73
Systematic review of management of incidental gallbladder cancer after cholecystectomy	Soreide, K	BRITISH JOURNAL OF SURGERYarrow_drop_down	2019	72
IRCAD recommendation on safe laparoscopic cholecystectomy	Conrad, C	JOURNAL OF HEPATO-BILIARY-PANCREATIC SCIENCES	2017	69
The risk of malignancy in ultrasound detected gallbladder polyps: A systematic review	Elmasry, M	INTERNATIONAL JOURNAL OF SURGERYarrow_drop_down	2016	69

The trends in these highly cited papers focus on the advancement and optimization of minimally invasive surgical techniques, improvements in diagnostic methods, the application of artificial intelligence, surgical safety, and retrospective studies. These research directions indicate that future treatment of gallbladder cancer will increasingly rely on the application of advanced technologies and comprehensive evidence analysis, aiming to improve surgical success rates and patient quality of life.

## Discussion

### General information

In our study, we analyzed 479 publications in the field of gallbladder cancer surgery over the past decade, using quantitative analysis software such as CiteSpace and VOSviewer to review the findings and developments. We conducted a quantitative analysis of basic information, including annual publication numbers, countries, institutions, disciplines, and journals. This study primarily focuses on research published in the field of gallbladder cancer surgery. To our knowledge, this is the first bibliometric and visualization analysis specifically examining the research status and development trends of publications related to gallbladder cancer surgery. This approach allows for a more precise and intuitive display of the research hotspots and frontiers in this field. In this study, to enhance the quality and standards of the included publications, we only considered relevant publications from the WOSCC database between 2014 and May 2024. However, in other databases, as early as 2006, scholars in China had already focused on using laparoscopic techniques to treat early-stage gallbladder cancer (22), alongside analyzing and evaluating the effectiveness of laparoscopic surgery. Based on the number of publications in the field of gallbladder cancer surgery, 36 publications were published in 2014, and the overall trend shows an increase. The more a publication is cited, the higher the attention it or the field receives. As shown by the linear prediction curve in **Figure 2B**, the number of publications and citation frequency in this field will continue to rise, reflecting that more scholars are recognizing the numerous unresolved issues that require further research and discussion in this area.

By conducting a statistical analysis of the number of papers published by various countries/regions, we can identify the key countries/regions that have published a significant amount of literature related to gallbladder cancer surgery and assess their impact, as well as determine their collaborative relationships. China, the United States, South Korea, Japan, and India are the main countries with the highest number of publications, with China ranking first. This may be related to the high burden of gallbladder cancer in Asia (primarily in terms of incidence and mortality), with China and India being particularly prominent (23). In the collaboration network, the United States serves as a core node, maintaining strong collaborative relationships with countries such as South Korea, Japan, and India. We also found that although China has published the most papers, it has relatively few collaborations with other countries/regions, which may hinder the long-term development of academic research. Although there are collaborative relationships between most countries, the breadth and strength of cooperation between institutions are not ideal. By filtering the top ten organizations based on link strength, the institutions with the most publications are Seoul National University, Yonsei University, and Sungkyunkwan University in South Korea. Moreover, as major institutions in South Korea, these organizations also have higher collaboration frequencies with each other compared to other institutions. There is only limited collaboration between numerous institutions in the United States and China, which also reflects that most studies in this field are predominantly single-center research (13, 24, 25). Close collaboration and communication between countries and institutions are beneficial for overcoming academic barriers and furthering research related to gallbladder cancer surgery.

Among the journals with the most publications in the field of gallbladder cancer surgery, few have high impact factors. The journals with the highest publication volumes, Surgical Endoscopy and Other Interventional Techniques (IF: 2.4) and Annals of Surgical Oncology (IF: 3.4), have relatively low impact factors. Although these journals are influential in this field, the majority of publications are retrospective clinical studies, which are limited by small sample sizes and low levels of evidence. Compared to basic research in oncology (26–28), it is challenging to achieve breakthrough advancements or significant academic impact in this area. Therefore, prospective multicenter studies are the direction in which breakthroughs in this field are likely to occur. Among the most cited articles, “Long-term outcomes after endoscopic ultrasonography-guided gallbladder drainage for acute cholecystitis” (29), published in Endoscopy with an IF of 11.5, has been cited 117 times. Although this article is not primarily focused on gallbladder cancer surgery, it discusses the application and long-term outcomes of endoscopic ultrasonography-guided gallbladder drainage (EUS-GBD) in patients with acute cholecystitis, including some patients with advanced gallbladder cancer who could not undergo curative surgery. EUS-GBD, as a drainage method, demonstrated high technical and clinical success rates, with favorable long-term outcomes. This is particularly important for patients with advanced gallbladder cancer, as they may not be able to tolerate traditional surgical treatments due to the cancer. This technique not only provides a safe and effective method for managing acute cholecystitis, but also significantly improves the quality of life for patients with advanced gallbladder cancer through high success rates and long-term stent patency (30), reducing the need for re-intervention and treatment-related discomfort, thus having significant clinical relevance.

Surgical Treatments of Hepatobiliary Cancers is a review published in 2021 in Hepatology with an IF of 12.9, and it has been cited 103 times (31). The review mainly discusses the postoperative management of incidental gallbladder cancer, the extent of surgery for T2 or lower stages, the role of lymph node dissection, the analysis of postoperative survival rates for T3 and T4 tumors, independent prognostic factors, and related controversial topics such as neoadjuvant and adjuvant therapies. Artificial Intelligence for Intraoperative Guidance Using Semantic Segmentation to Identify Surgical Anatomy During Laparoscopic Cholecystectomy is an article published in 2022 in Ann Surg with an IF of 7.5, cited 97 times. This study does not specifically address gallbladder cancer surgery, but rather focuses on using artificial intelligence for intraoperative guidance during laparoscopic cholecystectomy, utilizing semantic segmentation to identify surgical anatomy. The study (32) demonstrates that artificial intelligence (AI) performs well in identifying complex and poorly defined anatomical structures, providing potential technical support for quality improvement in future surgeries. AI has already been applied in research related to gastrointestinal tumors (33) and other fields (34, 35). However, its application in the field of gallbladder cancer remains unexplored, making the integration of AI technology a burgeoning area of interest among scholars.

### Hotspots and frontiers

Analysis of high-frequency keywords reflects the hotspots in a specific research field. By using keyword co-occurrence analysis, the main directions and hotspots in the field of gallbladder cancer surgery were identified, revealing the development and changes in gallbladder cancer surgery (36). In the keyword co-occurrence network, the color changes and bursts of keywords highlight the shift in research hotspots from early themes such as “laparoscopic cholecystectomy” and “experience” to more recent ones like “management,” “outcome,” and “radical cholecystectomy.” This indicates that the focus of research has shifted from the surgery itself to postoperative management, patient survival, recurrence, and the achievement of R0 resection. This reflects that patient benefit has gradually become the central focus and objective of most studies. According to the keyword timeline viewer, the research can be divided into three stages: Early research (2014-2016): During this period, research mainly focused on basic surgical techniques and disease classification, such as gallbladder cancer, cholelithiasis, and gallbladder disease. These keywords indicate that the research primarily concentrated on the causes and triggers of diseases (37), with an emphasis on early detection and prevention to reduce the incidence of gallbladder cancer. Mid-stage research (2017-2019): As technology advanced, research gradually shifted towards more complex and refined surgical techniques (38). For example, keywords such as laparoscopic surgery and hepatectomy (39, 40) began to appear more frequently, indicating that the use of laparoscopic techniques during malignant tumor surgeries had become a focal point of scholarly discussion. Precision surgery also gradually became a research hotspot: techniques such as ultrasonography (US), EUS-guided fine-needle aspiration (EUS-FNA) for preoperative tumor staging and lymph node assessment, intraoperative near-infrared fluorescence (NIRF) imaging using indocyanine green (ICG) for visualizing tumors and the biliary tree, and lymph node dissection (41), as well as enhanced recovery after surgery (ERAS) protocols to reduce hospital stay and complication rates (42). The significant increase in research on these techniques after 2017 suggests that, as surgical resection remains the only effective treatment, precise preoperative evaluation, meticulous intraoperative procedures, and postoperative management are crucial in improving prognosis. The widespread adoption of these techniques has made surgeries safer, less invasive, and quicker to recover from. Recent research (2020-2024): In recent years, advanced surgical techniques such as robotic surgery and minimally invasive surgery have become research focal points, reflecting the rapid advancement and application of medical technology. The main focus has been on whether minimally invasive surgery can offer benefits in terms of long-term survival and recurrence rates compared to open surgery. In fact, many studies comparing different surgical approaches generally favor the laparoscopic approach. However, these studies often have limited impact due to small sample sizes, single-center designs, and less rigorous methodologies (43, 44). What needs to be focused on regarding occult gallbladder cancer is that, firstly, chronic irritation of the gallbladder mucosa may lead to inflammation, replacement of the muscular layer, and loss of contraction, resulting in complications such as perforation or abscess, with occult tumors ultimately detected through histological examination. Another more insidious issue is porcelain gallbladder, characterized by calcification of the gallbladder wall, lack of contraction, and absence of pain, often leading to cancer development due to long-term chronic inflammation. Meanwhile, the increased frequency of keywords such as incidental gallbladder cancer and follow-up reflects growing attention to surgical outcomes and long-term health management. This underscores the emphasis in the medical field on improving patient quality of life and long-term health outcomes. The appearance of keywords like feasibility indicates that research is not only focused on applying existing technologies but also on exploring and validating new techniques and methods. This shows that researchers are continually seeking more effective and safer surgical methods and treatments. The shift from basic surgical techniques and disease management to more complex and advanced surgical technologies, with an increasing focus on long-term surgical outcomes and postoperative management, is evident. These trends reflect the rapid development of medical technology and its deepening clinical application, providing important reference points for future research and clinical practice.

In the diagnosis methods of gallbladder cancer, epidemiological studies, risk assessment, management of acute cholecystitis, application of laparoscopic cholecystectomy and related techniques, these keywords reveal researchers’ attention to the early diagnosis and surgical treatment of gallbladder cancer. Traditionally, ultrasound (US) has been the first-line imaging technique of choice for suspected gallbladder diseases. The imaging features of various benign gallbladder diseases are often similar to those of gallbladder cancer (45). As a result, gallbladder cancer is often difficult to differentiate from conditions such as xanthogranulomatous cholecystitis (46) and gallbladder polyps, leading to its detection at an advanced stage. At this stage, surgical treatment becomes more challenging, with poorer prognosis and significantly reduced postoperative benefits for the patient. Additionally, some patients are diagnosed with gallbladder cancer incidentally during laparoscopic surgery for benign gallbladder diseases. Thus, the precision of laparoscopic procedures can significantly impact the prognosis and survival of patients with incidental gallbladder cancer.

There are hot spots in the overall treatment strategy, chemotherapy regimen, surgery (especially lymph node dissection), metastasis mechanism and prognosis evaluation of gallbladder cancer, which reflects the attention of researchers to the treatment strategy and prognosis evaluation of gallbladder cancer. For different stages of resectable gallbladder cancer, there are some differences in the recommended surgical procedures in different guidelines (8). In various studies, this lack of uniformity in surgical techniques has led to slight differences in survival and recurrence rates (13, 47). Lymph node metastasis is one of the key factors determining prognosis. Lymph node dissection, particularly the removal of five or more lymph nodes, can provide a significant survival advantage, with survival rates markedly better than those of patients who do not undergo lymph node dissection (8). Even with lymph node dissection, patients remain at a high risk of recurrence, making postoperative chemotherapy and adjuvant therapy particularly important. In a large retrospective study, patients who received adjuvant therapy after surgery had fewer complications and a higher likelihood of lymph node positivity and positive surgical margins compared to those who underwent surgery alone. Therefore, in cases where lymph node involvement is uncertain, postoperative adjuvant therapy can improve overall survival rates (48).

Key words such as surgical methods of gallbladder cancer, postoperative prognosis assessment, patient survival rate, surgical guidelines, and meta-analysis reveal researchers’ concern about how to improve the prognosis of patients with gallbladder cancer through surgical resection, especially involving the application of laparoscopic radical surgery and the evaluation of its effect. One of the major points of contention in gallbladder cancer surgery is the surgical approach and the optimal extent of resection for early-stage gallbladder cancer. For example, the American Joint Committee on Cancer 8th Edition (AJCC 8th Edition) and the National Comprehensive Cancer Network (NCCN) guidelines both recommend radical resection for T1b gallbladder cancer. In contrast, the Chinese *Guidelines for the Diagnosis and Treatment of Gallbladder Cancer (2019 Edition)* suggest that patients undergo cholecystectomy plus wedge resection of liver tissue at least 2 cm from the gallbladder bed, along with regional lymph node dissection. For early-stage gallbladder cancer, surgical guidelines differ on the extent of intraoperative resection, and some meta-analyses suggest that there is no significant difference in overall survival outcomes between patients undergoing simple cholecystectomy and those undergoing extended cholecystectomy (49). Differences in the anatomical drainage pathways between T2a and T2b tumors result in variations in overall survival rates (50, 51), which in turn influence the choice of surgical approach. Additionally, analyses of survival benefits from laparoscopic radical cholecystectomy for gallbladder cancer show that, compared to open surgery, laparoscopic surgery offers better short-term benefits, such as reduced intraoperative bleeding, less postoperative pain, earlier resumption of eating, lower risk of wound infection, and shorter hospital stays (52, 53). Moreover, there is no significant difference in long-term survival outcomes (including survival and recurrence rates) among patients at different stages (24, 52, 54, 55).

The management and treatment of gallbladder cancer after operation, re-resection of residual lesions after operation, and surgical management strategies have also been paid attention by most scholars, because of the high probability of postoperative recurrence. Different guidelines recommend varying follow-up protocols. For example, the NCCN guidelines recommend imaging every 3-6 months during the first 2 years post-surgery, followed by every 6-12 months for up to 5 years. In contrast, the IHPBA expert consensus suggests that patients who did not receive adjuvant therapy undergo CT scans of the chest, abdomen, and pelvis every 3-4 months for 3-5 years (56, 57). Even when gallbladder cancer patients achieve an R0 resection, postoperative recurrence remains common. Once recurrence occurs, the prognosis is generally poor; even with multidisciplinary treatments, including re-surgery or chemoradiotherapy, the 5-year overall survival rate remains only 15-20% (58).

Similarly, the surgical treatment experience of gallbladder cancer and occasional gallbladder cancer, specimen management, and the transformation of minimally invasive and open surgery are also hot topics. Gallbladder cancer is a rare but highly aggressive cancer, with most cases being incidentally discovered after cholecystectomy. A study from Gold Coast University Hospital found that 0.46% of 3,904 cholecystectomy specimens were diagnosed with gallbladder cancer (GBC), with half of these cases being incidentally discovered postoperatively. Female gender, higher BMI, and increased age were identified as risk factors for GBC (59). In the past, when laparoscopic techniques were less developed, preventing specimen rupture and port-site implantation metastasis during removal was a clinical concern, whether for incidental or non-incidental gallbladder cancer. However, with the widespread use of specimen retrieval bags, meta-analyses have shown that the rate of port-site implantation metastasis after laparoscopic surgery for Tis-T2 gallbladder cancer has decreased from an early range of 10%-18% to 1.3% (60). Gallbladder cancer is highly malignant, and surgery is technically challenging, with often only one chance for curative treatment. Regardless of the treatment method chosen, the primary goal should be to maximize patient benefit. Furthermore, I believe that the concept of enhanced recovery after surgery (ERAS) should not be equated with a purely laparoscopic approach. If complications such as increased surgical complexity, peritoneal dissemination, or incomplete clearance arise, open surgery is recommended; otherwise, the patient may face a poor prognosis, rendering the short-term benefits of laparoscopy irrelevant.

### Limitations

In this study, to ensure high-quality bibliometric analysis, the analysis was based on articles from the WoSCC database, leading to the omission of many studies published in non-SCI journals or other databases. Secondly, since citation data is time-dependent, more recently published articles may have fewer citations compared to earlier ones, primarily due to the limitation of publication dates. These limitations may slightly affect the overall results but are unlikely to significantly impact the main trends in the research field. Finally, due to insufficient data, not all publications from 2024 could be included.

## Conclusions

This study is the first bibliometric analysis in the field of gallbladder cancer surgery. Through bibliometric and visual analysis, this study revealed the research trends and hotspots in the field of gallbladder cancer surgery and identified future research directions. Future research should further strengthen international collaboration, deepen the study of minimally invasive surgery and postoperative management, and actively explore the application of emerging technologies in gallbladder cancer treatment, with the aim of continuously improving patient outcomes and quality of life.

## Data Availability

The original contributions presented in the study are included in the article/supplementary material. Further inquiries can be directed to the corresponding authors.
